# Developing a dementia inclusive hospital environment using an Integrated Care Pathway design: research protocol

**DOI:** 10.7717/peerj.11589

**Published:** 2021-07-14

**Authors:** Jorge Riquelme-Galindo, Manuel Lillo-Crespo

**Affiliations:** 1 Nursing Management, HLA Vistahermosa Hospital, Alicante, Spain; 2 Faculty of Health Sciences, Universidad de Alicante, Alicante, Spain

**Keywords:** Dementia, Hospitals, Nursing, Standards of care, Evaluation, Integrated care pathway

## Abstract

People with dementia occupy 25% of the hospital beds. When they are admitted to hospitals their cognitive impairment is not considered in most of the cases. Some European and North American countries already have experience of implementing national plans about Alzheimer’s disease and dementia. However South European countries such as Spain are in the early stages. The aim of this study is to design an Integrated Care Pathway to adapt the hospital environment and processes to the needs of people with dementia and their caregivers, generating a sense of confidence, increasing their satisfaction and protecting them from potential harmful situations. This study uses King’s Fund Dementia Tool to assess the hospital environment and develop a continous improvement process. People with dementia, families, caregivers and healthcare staff will evaluate the different settings in order to provide guidance based on patient needs. Person-centred care, prudent healthcare and compassionate care are the conceptual framework of this care pathway. The implementation and evaluation of this research protocol will provide information about how to successfully design dementia interventions in a hospital environment within available resources in those contexts where dementia plans are in its infancy, as only around 15% of all states worldwide have currently designed a concise dementia national plan.

## What is the main question being addressed in your study?

Dementia population data predictions worldwide are alarming and the International Alzheimer Association estimates that 75.6 million people in 10 years since 2020 will suffer dementia reaching 135.5 millions in 2050 (Prince et al., 2016). In line with this, several worldwide healthcare systems have for years implemented specific dementia national plans (World Health Organization, 2017) while Spain is still in its early stages with its national Alzheimer and dementia Plan for the period 2019–2023 (Ministerio de Sanidad, Consumo y Bienestar Social, 2019). At the moment of this study, Spain is still not included as a country with an established national dementia plan, only with a National Alzheimer Strategy. The number of worldwide countries following Alzheimer’s Disease International guidelines are only 27 out of 194 (13%), which means that Spain is included in the other 87% of the nations that still do not meet criteria (Alzheimer Disease International, 2020).

Spain is one of the countries with more life expectancy worldwide and the first one in Europe (Organisation for Economic Cooperation & Development & World Health Organization, 2019). More than 57.1% of the Spanish hospital beds are occupied by old adults (Abellán García et al., 2019) and at least 25% of them are people living with dementia (Alzheimer’s Society, 2016). Regarding dementia care in Spain, it is necessary to remark on its current transition situation. Traditionally, it has been assumed that the caregiving role of the Spanish older population corresponds to the family, concretely to women (Lillo-Crespo et al., 2018). Currently, it is changing from being cared at home to more institutionalised care, as in the last two decades the number of older people living in residences and care homes has also grown more than three times in the country (Lillo-Crespo & Riquelme, 2018). However, specific initiatives for such a Spanish population have not been implemented in hospitals and healthcare centres until now and consequently this situation challenges health organizations nationwide to be equipped with adapted environments and trained human workforce. Moreover, strategies need to be taken in different contexts earlier than expected due to COVID-19 pandemic situation and global economic recession, as national health budgets will be affected (Organisation for Economic Cooperation & Development, 2020). The main question being addressed in our study is focused in how to transform hospital settings in a dementia-friendly environment.

Hospitalization have shown to be a challenging time for patients whose brain impairment will make it difficult for them to adapt to an unfamiliar environment and unknown people, such as the healthcare staff (Kazui et al., 2004). Cost-related dementia people hospitalisation is estimated to be almost three times more when compared with non-dementia patients (Briggs et al., 2016), and people living with dementia have almost double the hospital stays per year than any other older people (Shepherd et al., 2019). Clinical pathways and Integrated Care Pathways (ICP) have been designed for more than 30 years in different contexts with the purpose to enhance the quality of care across the continuum by improving risk-adjusted patient outcomes, promoting patient safety, increasing patient satisfaction, and optimizing the use of resources as described by Schrijvers, Van Hoorn & Huiskes (2012).

Thus, the previous step to start this research was to explore the status quo of the research question of this study through a Scoping Review. Two different literature searches about the topics Care Pathway and Dementia Friendly Hospital Adaptation were done. Parallel, a grey literature review complemented the information collected. Person-centred care, prudent healthcare and compassionate care were the components that emerged from the review as strong themes. They were found as the core values of dementia care, in relation with gold standards of care and management. This care pathway will include these key elements as a framework during the process of design and implementation. In line with the results of the scoping review conducted our dementia pathway will be designed around the importance of: the context or environment, the person’s and families experience, and the neurological impairment condition of dementia. Kitwood (1997) believed the environment can influence the brain as much as the brain influences the person’s abilities. Kitwood & Bredin (1992) agreed that dementia syndromes are manifested differently from person to person due to the context they belong to and live in though they found a common need in people living with dementia that implies recognition, respect and trust that emphasises personhood. From a conceptual positioning, our study takes references from the concept of dementia-friendly hospital developed by Parke et al. (2014) linked to the four dimensions associated with the author’s foundational previous work on elder-friendly hospitals: (1) social climate; (2) policy and procedures; (3) care systems and processes; and (4) physical design.

Dementia-friendly environment interventions have helped to design adapted environments for people living with dementia taking into account their experiences, common behaviours, habits and enablers to facilitate their daily routines (Davis et al., 2009). These interventions have contributed to tackle the stigma of health organizations as unknown and dangerous environments. Different dementia-friendly environment initiatives have been successfully implemented in regions known for being sensitive with this population as it is the case of North European, North American and Canadian countries where several norms, laws, policies and strategies to support people living with dementia are established (Quasdorf et al., 2017; Biglieri, 2018). Furthermore relevant studies have been published where people living with dementia and healthcare staff are included in decision-making about dementia hospitals adaptations (Hung et al., 2017; Hung, Son & Hung, 2019; Clifford & Doody, 2018).

From our conceptual perspective the combination of Compassion and person-centred care is the gold standard while caring of people living with dementia (Kitwood, 1997; Behuniak, 2010) even though it is not always prioritized in Health organizations (Dewing & Dijk, 2016). Compassionate care perspective is the dynamic process of recognising the uniqueness of each individual and therefore it is characterised for being person-centred. Compassionate care implies having the disposition and acting attitude based on the knowledge, emotions, strengths, intuitions, concerns and suffering of the patient, thus involving the professional in care. Moreover, it leads health professionals to act with warmth, individualized attention according to the unique circumstances of the patient and its family (Sinclair et al., 2017). In fact, more than nine over 10 people living with dementia in England answered in a survey that they found the hospital environment as “frightening” (Alzheimer’s Society, 2016).

However trying to implement a new care culture in a healthcare organisation may bring economic aftermaths. The paradigm named as Prudent Healthcare has been used in literature when talking about efficiency in dementia Care (Tolson et al., 2016). Prudent Healthcare is defined as healthcare which is conceived, managed and delivered in a cautious and wise way characterised by forethought, vigilance and careful budgeting which achieves tangible benefits and quality outcomes for patients (Hughes, 2016). It aims to address the needs of the patient and avoid ineffective care provided that it does not produce a patient benefit.

Thus our study is focused on the three key elements regarding inclusion, management and care of people living with dementia and family in healthcare settings. Compassion and person-centred care with a Prudent Healthcare management perspective may contribute to adapt clinical environments to people living with dementia and their caregivers’ needs. This study can be used as an example for those healthcare settings where dementia-friendly initiatives have not been implemented yet.

## Describe the aims, context and preliminary methods

This study takes place in the Southeast coast of Spain, in the province of Alicante where 20.9% of the population are 65 years old and over. The Spanish mean of this population corresponds to 19.58%. Hence Alicante province is above the average of Spanish provinces in terms of old population (Instituto Nacional de Estadística, 2020) representing an outstanding number of people that could potentially suffer of dementia. The total amount of inhabitants in the city of Alicante (the main city of the province) and surroundings in 2020 are more than 400.000 people. Alicante province is well known as a living area for retired older people mostly arriving from the Centre and North of Europe that increases every year the number of people with 65 years and over representing the 18% of the total amount of the province.

The chosen setting to design our ICP is a hospital environment specifically a private hospital called HLA Vistahermosa that belongs to a 15-hospital group called Hospitales Lavinia Asociados (HLA) which is considered as a reference in the clinical attendance of national and international patients. It is located in the city of Alicante and it is 100-beds size usually admitting around 53 patients with a diagnosis of dementia per month to the Emergency Room and the hospitalisation services (own data extraction calculating the yearly mean of hospitalised patients with dementia in HLA Vistahermosa Hospital).

Before starting with the dementia ICP design and implementation, we aimed to evaluate how much the environment and practices were adapted for people living with dementia. Our pilot test was based on the King’s Fund Evaluation tool “Is your hospital dementia friendly?” EHE Environmental Assessment Tool (The King’s Fund, 2014). It was selected as it is the mostly used tool in European healthcare contexts for evaluating dementia Hospital settings and came up as part of different publications during the scoping review carried out earlier in this project. This tool potentially provides data collected not only from the surveys but also from focus groups (Waller & Masterson, 2015). Permissions were requested from the authors although they were not required since it is an open access tool. The evaluation took place along the first quarter of 2020 after receiving the approval and consent from the hospital and the 130 health professionals participating .

Participants were asked throughout an online platform to give consent. Information about professional role and years of experience were asked. Information provided by the questionnaire platform was the following: *The personal data provided by you will be treated in accordance with the General Data Protection Regulation (GDPR 2016/679) and Spanish Organic Law 3/2018, of December 5, 2018, for the purpose of managing your participation in the study. Those interested have the right to request the data controller for access, rectification, deletion, portability of their data, as well as the right to limit and oppose treatment. If you want to exercise any of the aforementioned rights, please write to jorge.riquelme@grupohla.com*.

The King’s Fund evaluation tool was completed by 130 healthcare full-time professionals who had been working at HLA Vistahermosa Hospital for at least 7 years. Participants were nurse-assistants, nurses and physicians from different areas (Emergency Room, Intensive Care Unit, Hospitalization, Surgical Area and outpatient services) all of them skilled in reading and understanding English. According to the structure of the tool our environment was evaluated from seven perspectives: (1) interaction between others, (2) wellbeing, (3) active engagement in own care, (4) mobility, (5) personal hygiene, (6) orientation, (7) calm, safety and security. Respondents answered each question individually by using numbers ranging from 1 to 5 (1 = barely met, 5 = totally met). After collecting data, focus groups were carried out in order to obtain more qualitative information about the answers provided. Sixteen Professionals considered as key informants were invited to discuss, divided in two groups, about the current situation of the hospital and analysed the positive and negative aspects. Focus groups took place in different days at the same meeting area during 60 min each one. They were moderated by the authors of this study and the conversation guideline was the different perspectives from the questionnaire mentioned before. Participants were asked to give their opinions and discuss about the positive and negative aspects regarding the current environmental adaptation of the hospital to people living with dementia.

Most of the participants were nurses (70%), while nurse assistants (20%) and physicians (10%) represented a lower proportion. For the evaluation tool analysis we interpreted that 100% meant “totally met” and 0% meant “barely met”. Table 1 shows the results obtained. The average obtained was 68.89%.

**Table 1:** Results from evaluating hospital environment.

Position	CRITERION	PERCENTAGE
1	The environment promotes well-being	77.72%
2	The environment promotes meaningful interaction between patients, their families and staff	74.2%
3	The environment promotes calm, safety and security	73.2%
4	The environment promotes continence and personal hygiene	71.12%
5	The environment encourages eating and drinking	66.67%
6	The environment promotes mobility	60.26%
7	The environment promotes orientation	59.07%
	TOTAL	68.89%

During the two focus groups, healthcare staff focused on the areas they belonged to and their own experience. Some of the ideas discussed were reported as unique initiatives conducted by one person or a small group of people though not as a mandatory task or part of one specific protocol. The Table 2 includes some of the most relevant remarks classified by positive and negative remarks:

**Table 2:** Positive and negative remarks from focus groups.

The King’s fund themes	Remarkable comments (negative & positive)
1. The environment promotes well-being	*1.“Lack of awareness in the surgical area to identify people living with dementia and avoid use of drugs that can potentially interact with post operative wellbeing”*. *2. “Visit restraints, lack of natural light, lack of daily routines adaptation and the few use of music therapy were identified”*.
2. The environment promotes meaningful interaction between patients, their families and staff	*1. “Hospitalisation bedrooms are individual and count with an extra bed so families and caregivers can stay 24/7 involving them in daily care activities of patients”*. *2. “Lack of inclusion of families and caregivers in the surgical area”*.
3. The environment promotes calm, safety and security	*1. “Noise and disturbances could be better controlled during transfer periods as corridors sometimes are noisy areas”*. *2. “Physical and Pharmaceutical retraints protocol facilitate the late use of these measures to control People living with dementia symptoms”*.
4. The environment promotes continence and personal hygiene	*1. “Single patient bedroom in hospitalisation facilitates privacy for personal hygiene”*. *2. “Hospitalisation area counts with bedrooms that include single WC, shower and bathroom sink”*.
5. The environment encourages eating and drinking	*1. “Patients and families are asked for their preferences during their stay in terms of nutritional habits”*. *2. “Families and caregivers are empowered to collaborate with healthcare staff during meals”*.
6. The environment promotes mobility	*1. “No open areas further than corridors or bedrooms promote mobility”*. *2. “Physiotherapist, nurses and nurses assistants collaborate with families in order to promote mobility during patient hospital stay”*.
7. The environment promotes orientation	*1. “Corridor bedroom number identification was confusing and not easy to use for wayfinding”*. *2. “All corridors at different floors are similar in colours and design so it is difficult for wayfinding”*. *3. “Wayfinding signals with big letters where not found and colours used do not contrast so reading must be difficult for people living with dementia”*. *4. “Single patient bedroom reduces agitation and confusion episodes”*.

After analysing the results obtained with The King’s fund evaluation tool from healthcare professionals, it was demonstrated that this hospital promotes wellbeing, contributes with the interaction between families and staff and respects the privacy of the people living with dementia. However, orientation and mobility was not valued as highly scored as the other items. The selected tool contributed to define the key elements that need to be adapted when referring to hospital environment to maximise those items identified as positive aspects and support or change those valued as negatives ones from a context-based perspective.

Thus the intervention of this study will be focused in developing an Integrated Care Pathway following King’s Fund rationales mentioned before, stressing in the areas professionals identified as weaker compared with the other ones. An Integrated Clinical Pathway Checklist (Whittle et al., 2004) will be used to ensure that the structure follows reference guidelines to design ICP, that the mechanism used is robust, and that the ICP documentation meets at least the minimum legal requirements for clinical documentation. In addition, patients and families will complete at the end of their stay “Is your hospital dementia friendly? EHE Environmental Assessment Tool—King’s Fund” in order to evaluate the impact of the ICP implementation in clinical settings.

## What are your research questions and hypotheses?

The main research question of this project is focused in how to transform hospital settings in a dementia-friendly environment. The conceptual hypotheses of this qualitative research corresponding with the study aim are:

- Hypothesis 1: changes in hospital environment based on the King’s Fund assessment tool may have a positive impact on people living with dementia and families perception of the hospital environment as dementia-friendly.
- Hypothesis 2: staff training to improve compassionate and person-centred care may have a positive impact on people living with dementia and families perception of the hospital environment as dementia-friendly.

## How many and which conditions will participants/samples be assigned to?

In a previously published study based on a European Commission funded project named as Palliare in which authors participated, some of the most typical cases of people living with dementia in Europe were described (Lillo-Crespo et al., 2018) including the case of Spain as shown in Table 3. Considering the context that we are exploring in this study, we are aiming to collect information about those critical cases identified as representative in clinical settings.

**Table 3:** Demographic data from representative cases.

Case	Main caregiver gender	Years since diagnosis	Settings where PwD is cared	Age of PwD
1	Female	5	Residence	83
2	Female	3	Relative’s home/day centre	80
3	Male	1	Own home	64

There will be three different study groups, (1) patients and (2) families/caregivers (3) healthcare staff selected according to their representativeness. The first one corresponds to people diagnosed of dementia attended at the emergency room as well as those hospitalised for any reason. The second group will be those who spend more time in caring the patient admitted and diagnosed with any type of dementia considered in the inclusion criteria. The third group will be the same healthcare staff that completed the preliminary survey conducted in order to check if they have found differences or not in the items studied with King’s Fund tool.

## How many observations will be collected and what rule will you use to terminate data collection?

The average of people living with dementia who visit the target hospital and are admitted to the ER and hospitalization due to any reason are around 53 patients per month.

We are aiming to collect information about the maximum number of cases that follow the inclusion criteria during a period of 3 months (October, November and December 2020) and then we will start designing the final ICP for the specific context selected.

The King’s Fund survey that evaluates the different seven perspectives about the hospital environment will be also asked to participants to be completed by the end of their stay in the Hospital. If there is any reason of not being able to answer the questionnaire it could be sent a maximum time of 15 days after hospital discharge by email.

In regard to healthcare staff completion of the King’s Fund survey, it will be conducted at the end of December 2020 in order to evaluate the performance carried out along the three months of training, design adaptation and implementation of changes based on the weaker areas identified in the previous evaluation. Later, the final ICP will be shared and implemented in other hospitals from the HLA hospital group across the country .

## What are your study inclusion criteria?

In the case of participants with dementia, researchers followed ethical recommendations of Alzheimer Europe (2011), and the WHO (Cash et al., 2009) as there is a lack of national guidance in Spain. Following recommendations of the previously cited organisations, the surveys with individuals living with dementia will always be conducted with the legally responsible family member being present: (a) questions will be addressed directly to the family member, although the person with dementia is present and could express their opinions, and (b) the survey will take place when the person with dementia is oriented and cognitively able. During the surveys, interviewers have to be attentive to signs of distress linked to participation, checking whether the participant wished to withdraw.

Participants of group 1 will be patients admitted to HLA Vistahermosa Hospital within October, November and December 2020 period.

Group 1 (Patients):

- Those able to respond the King’s Fund questionnaire with of Glasgow scale (to evaluate the alert level of the patient) value of 15 and/or Pfeiffer scale (to evaluate the level of cognitive impairment) score higher than 2.
- People diagnosed with any of dementia types independently of the time: Alzheimer’s, Vascular dementia, Lewy body dementia, Parkinson’s, Frontotemporal, Creutzfeldt-Jakob, Wernicke-Korsakoff Mixed dementia.

Group 2 (Family/caregiver):

- Those able to understand and respond the questionnaire
- Those that are experienced for caring people with dementia for at least 1 month.

Group 3 (Healthcare staff):

- Full time professionals such as nurse-assistants, nurses and physicians from Emergency Room, Intensive Care Unit, Hospitalization, Surgical Area and outpatient services with working experience of at least 7 years.

When referring to the questionnaire, only those fully completed will be included in the analysis and those sent with due date of 15th January 2021.

## What are your data exclusion criteria?

For the Group 1 (Patients)

- Those unable to respond the King’s Fund questionnaire with of Glasgow scale value lower than 15 and/or Pfeiffer scale score lower than 2.
- People diagnosed with any of dementia types independently of the time: Alzheimer’s, Vascular dementia, Lewy body dementia, Parkinson’s, Frontotemporal, Creutzfeldt-Jakob, Wernicke-Korsakoff Mixed dementia.

For Group 2 (Family/caregivers):

- Those unable to understand and respond the questionnaire.
- Those unexperienced for caring people with dementia for less than 1 month.

For Group 3 (Healthcare staff)

- Professionals not working in units such as the Emergency Room, Intensive Care Unit, Hospitalization, Surgical Area and outpatient services.
- Professionals less experienced than 7 years.
- Professionals who are not nurse-assistants, nurses or physicians.

Those uncompleted questionnaires won’t be included for analysis.

## What positive controls or quality checks will confirm that the obtained results are able to provide a fair test of the stated research question and hypothesis?

In order to build a comprehensive and context based ICP, the Integrated Clinical Pathway Checklist will be used to ensure that the pathway developed is an ICP, and the mechanism used to develop the ICP is structured, well documented, the process follows a scientific contrasted approach and the implementation process is completed. Moreover, towards ensuring that the care process is person-centred, The Care Process Self Evaluation Tool^©^ will be used (Seys et al., 2013). This evaluation tool will serve as a quality check to evaluate after the implementation how much (from 1 to 10) the organisation is patient focused, coordinated with other professionals, follows high standards of patient and family communication and counts with an existing follow-up process oriented to continuing improvement. The multidisciplinary team will evaluate their own organisation and performance taking into account the care process of dementia patients in January 2021.

## Specify exactly which analyses will be conducted to examine the main question/hypothesis(es)

As it has been mentioned before, we will analyse both hypotheses through EHE Assessment tool in order to collect information before and after implementing the Dementia Integrated Care Pathway. Special attention will be paid to those items identified by healthcare professionals in the pilot study as weaker. An independent analysis triangulation strategy will be conducted towards validating the qualitative data collected by three experienced researchers.

## Are you proposing to collect new data or analyze existing data?

**Figure 1:**
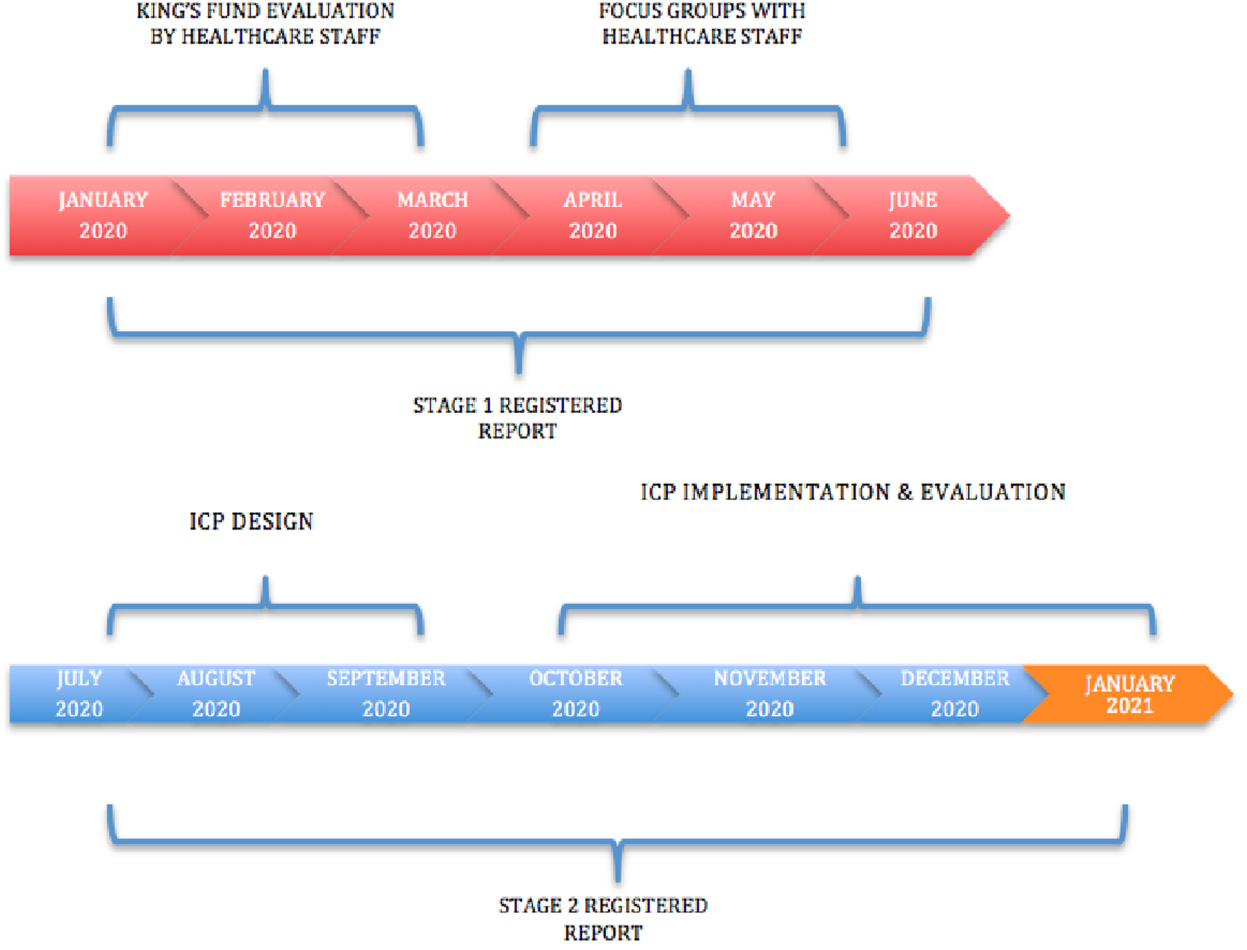
Timeline process.

Prospectively new data will be collected from people living with dementia, families and healthcare staff in different hospital settings towards guaranteeing that the ICP is context based. Once having evaluated all the contexts and adapted specifically the ICP an evaluation will be carried every two years as a part o the continuous evaluation and improvent process. Anonymous online or printed surveys will be conducted. The different phases of the project is shown in Fig. 1. The present study was approved by the HLA Hospital Group Management Committee and Research Ethics Board in September 2019.

## Additional Information and Declarations

### Data Availability

The following information was supplied regarding data availability: This article does not include raw data or code.

## References

[ref-1] Abellán García A, Aceituno Nieto P, Pérez Díaz J, Ramiro Fariñas D, Ayala García A, Pujol Rodríguez R (2019). Un perfil de las personas mayores en España, 2019 Indicadores estadísticos básicos.

[ref-2] Alzheimer Disease International (2020). Dementia plans. https://www.alzint.org/what-we-do/policy/dementia-plans/.

[ref-3] Alzheimer Europe (2011). Informed consent to dementia research: ethics of dementia research. http://www.alzheimer-europe.org/Ethics/Ethical-issues-in-practice/2011-Ethics-of-dementia-research/Informed-consent-todementia-research#fragment3.

[ref-4] Alzheimer’s Society (2016). Fix dementia care: hospitals.

[ref-5] Behuniak SM (2010). Toward a political model of dementia: power as compassionate care. Journal of Aging Studies.

[ref-6] Biglieri S (2018). Implementing dementia-friendly land use planning: an evaluation of current literature and financial implications for greenfield development in suburban Canada. Planning Practice & Research.

[ref-7] Briggs R, Coary R, Collins R, Coughlan T, O’Neill D, Kennelly SP (2016). Acute hospital care: how much activity is attributable to caring for patients with dementia?. QJM: An International Journal of Medicine.

[ref-8] Cash R, Wikler D, Saxena A, Capron AM, World Health Organization (2009). Casebook on ethical issues in international health research.

[ref-9] Clifford C, Doody O (2018). Exploring nursing staff views of responsive behaviours of people with dementia in long-stay facilities. Journal of Psychiatric and Mental Health Nursing.

[ref-10] Davis S, Byers S, Nay R, Koch S (2009). Guiding design of dementia friendly environments in residential care settings: considering the living experiences. Dementia.

[ref-11] Dewing J, Dijk S (2016). What is the current state of care for older people with dementia in general hospitals? A literature review. Dementia.

[ref-12] Hughes D (2016). UK-wide health policy under the Coalition. Dismantling the NHS?: Evaluating the Impact of Health Reforms.

[ref-13] Hung L, Phinney A, Chaudhury H, Rodney P, Tabamo J, Bohl D (2017). “Little things matter!” Exploring the perspectives of patients with dementia about the hospital environment. International Journal of Older People Nursing.

[ref-14] Hung L, Son C, Hung R (2019). The experience of hospital staff in applying the Gentle Persuasive Approaches to dementia care. Journal of Psychiatric and Mental Health Nursing.

[ref-15] Instituto Nacional de Estadística (2020). Proporción de personas de 65 y más años en España. https://www.ine.es/jaxiT3/Datos.htm?t=1488#!tabs-mapa,Accessed18.

[ref-16] Kazui H, Hashimoto M, Nakano Y, Matsumoto K, Yamamura S, Nagaoka K, Mori E, Endo H, Tokunaga H, Ikejiri Y, Takeda M (2004). Effectiveness of a clinical pathway for the diagnosis and treatment of dementia and for the education of families. International Journal of Geriatric Psychiatry.

[ref-17] Kitwood TM (1997). Dementia reconsidered: the person comes first.

[ref-18] Kitwood T, Bredin K (1992). Towards a theory of dementia are: personhood and well-being. Ageing and Society.

[ref-19] Lillo-Crespo M, Riquelme J (2018). From home care to care home: a phenomenological case study approach to examining the transition of older people to long-term care in Spain. Journal of Research in Nursing.

[ref-20] Lillo-Crespo M, Riquelme J, Macrae R, De Abreu W, Hanson E, Holmerova I, Cabañero MJ, Ferrer R, Tolson D (2018). Experiences of advanced dementia care in seven European countries: implications for educating the workforce. Global Health Action.

[ref-21] Ministerio de Sanidad, Consumo y Bienestar Social (2019). Plan Integral de Alzheimer y otras Demencias 2019–2023. https://www.mscbs.gob.es/profesionales/saludPublica/docs/Plan_Integral_Alhzeimer_Octubre_2019.pdf.

[ref-22] Organisation for Economic Cooperation and Development (2020). Coronavirus (COVID19): joint actions to win the war. https://www.oecd.org/about/secretary-general/Coronavirus-COVID-19-Joint-actions-to-win-the-war.pdf.

[ref-23] Organisation for Economic Cooperation and Development and World Health Organization (2019). State of Health in the EU Spain Country health profile 2019: European observatory on health systems and policies.

[ref-24] Parke B, Hunter KF, Bostrom AM, Chambers T, Manraj C (2014). Identifying modifiable factors to improve quality for older adults in hospital: a scoping review. International Journal of Older People Nursing.

[ref-25] Prince M, Comas-Herrera A, Knapp M, Guerchet M, Karagiannidou M (2016). World Alzheimer report 2016: improving healthcare for people living with dementia: coverage, quality and costs now and in the future. https://www.alzint.org/u/WorldAlzheimerReport2016.pdf.

[ref-26] Quasdorf T, Riesner C, Dichter MN, Dortmann O, Bartholomeyczik S, Halek M (2017). Implementing dementia care mapping to develop person-centred care: results of a process evaluation within the Leben-QD II trial. Journal of Clinical Nursing.

[ref-27] Schrijvers G, Van Hoorn A, Huiskes N (2012). The care pathway: concepts and theories: an introduction. International Journal of Integrated Care.

[ref-28] Seys D, Deneckere S, Sermeus W, Gerven EV, Panella M, Bruyneel L, Mutsvari T, Bejarano RC, Kul S, Vanhaecht K (2013). The Care Process Self-Evaluation Tool: a valid and reliable instrument for measuring care process organization of health care teams. BMC Health Services Research.

[ref-29] Shepherd H, Livingston G, Chan J, Sommerlad A (2019). Hospitalisation rates and predictors in people with dementia: a systematic review and meta-analysis. BMC Medicine.

[ref-30] Sinclair S, Beamer K, Hack TF, McClement S, Raffin Bouchal S, Chochinov HM, Hagen NA (2017). Sympathy, empathy, and compassion: a grounded theory study of palliative care patients’ understandings, experiences, and preferences. Palliative Medicine.

[ref-31] The King’s Fund (2014). Is your hospital dementia friendly?. London The King’s fund. https://www.kingsfund.org.uk/sites/default/files/media/ehe-hospitals-dementia-assessment-tool.pdf.

[ref-32] Tolson D, Fleming A, Hanson E, De Abreu W, Crespo ML, Macrae R, Jackson G, Hvalič-Touzery S, Routasalo P, Holmerová I (2016). Achieving prudent dementia care (Palliare): an international policy and practice imperative. International Journal of Integrated Care.

[ref-33] Waller S, Masterson A (2015). Designing dementia-friendly hospital environments. Future Hospital Journal.

[ref-34] Whittle CL, McDonald PS, Dunn L, De Luc K (2004). Developing the Integrated Care Pathway appraisal tool (ICPAT): a pilot study. Journal of Integrated Care Pathways.

[ref-35] World Health Organization (2017). Global action plan on the public health response to dementia 2017–2025. https://www.alzint.org/what-we-do/partnerships/world-health-organization/who-global-plan-on-dementia/#:~:text=The%20Global%20action%20plan%20on,Disease%20International%20and%20others%20worldwide.

